# Surgical resection of calcifying nested stromal-epithelial tumor in an adolescent female: A case report

**DOI:** 10.1016/j.ijscr.2019.11.018

**Published:** 2019-11-16

**Authors:** Nicholas Olin, Ankit Patel, Susan S. Baker, Roberto Hernandez-Alejandro

**Affiliations:** a University of Rochester, School of Medicine and Dentistry, Rochester, NY, 14642-0001, USA; b Department of Surgery, University of Rochester School of Medicine and Dentistry, Rochester, NY, 14642-0001, USA; c University at Buffalo Jacobs School of Medicine and Biomedical Sciences, Buffalo, NY, 14201-1520, USA

**Keywords:** Calcifying nested stromal-epithelial tumor, Liver neoplasm, Surgical management, Hepatectomy, ALPPS

## Abstract

**Introduction:**

Calcifying nested stromal-epithelial tumor (CNSET) is an extremely rare, indolent tumor of the liver of uncertain cellular origin. With only 38 cases reported in the literature, pathogenesis and optimal therapeutic approach are not well characterized. Based on the available literature, the risk of recurrence is low with surgical resection with negative margins.

**Presentation of case:**

In this case report, we describe an adolescent patient with CNSET who underwent right trisectionectomy.

**Discussion:**

In order to avoid posthepatectomy liver failure, special consideration must be given to the amount of residual liver parenchyma after resection when considering surgical approach. Single stage right trisectionectomy and two stage via associating liver partition and portal vein ligation for staged hepatectomy (ALPPS) were both considered for surgical approach in this case in order to provide sufficient functional liver parenchyma remnant.

**Conclusion:**

Given that obtaining negative margins is important in reducing the risk of recurrence, the method of surgical resection utilized is based on the amount of future functional residual hepatic parenchyma.

## Highlights

• Calcifying nested stromal-epithelial tumor is an extremely rare tumor of the liver.
• Surgical resection with negative margins has demonstrated low risk of recurrence.
• Surgical approach is dependent upon adequate future liver remnant.
• ALPSS should be utilized when resection would leave inadequate liver remnant.

## 1 Introduction

Calcifying nested stromal-epithelial tumor (CNSET) is an extremely rare tumor of the liver first described in 2001. It is characterized by nests of epithelioid and spindle cells with a desmoplastic myofibroblastic stroma and variable calcification and ossification. There have been 38 cases reported in the literature, with the tumor occurring most frequently in women (male to female ratio 1:2.5) [1]. In the cases reported, CNSET was often incidentally found in asymptomatic patients [2]. Symptomatic patients present with abdominal mass, abdominal pain, and there have been several cases reported of presentation with Cushing syndrome [3]. There are possible associations with Beckwith-Weidemann and Klinefelter syndrome [4,5]. Treatment is surgical resection or transplantation if resection is not feasible. Patients who undergo surgical resection most often remain disease free throughout follow up. However, there have been a few cases of recurrence reported. In this report, we describe a 15 year old patient with CNSET who underwent surgical resection of the tumor. This report has been written in accordance with SCARE criteria guidelines for case reports [6].

## 2 Case report

A 15 year old athletic female with past medical history of asthma presented to the emergency department with a 4 day history of acute onset right abdominal pain and shortness of breath. She had been in her usual state of health prior to this, maintaining an active lifestyle. She did not have significant change in weight or appetite. She did not endorse nausea, vomiting, fever or chills.

The patient had no history of tobacco, alcohol, or illicit drug use. The results of laboratory workup were unremarkable. It revealed an ALT 12 U/L, AST 19 U/L, Alkaline Phosphatase 84 U/L, TB 0.2 mg/dL, INR 0.94 and an Albumin 4.6 g/dL.

**Fig. 1:**
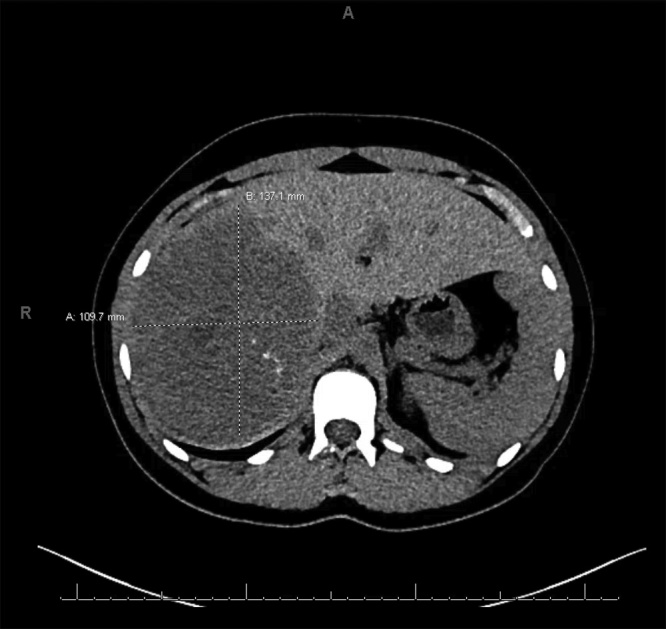
Triphasic abdominal CT scan of patient’s abdomen with CNSET indicated.

Radiographic imaging was obtained at her initial presentation. An abdominal ultrasound revealed a large heterogenous mass in the liver. An abdominal computerized tomographic (CT) scan revealed a mass with scattered calcifications and a central non-enhancing region (10.8 × 13.7 × 12.8 cm) in the right hepatic lobe predominantly in segments 7 and 8, along with ovarian cysts. A triphasic scan showed a hypodense lesion which demonstrated heterogenous enhancement on arterial phase imaging (Fig. 1). No obvious invasion of the vasculature, extension in the biliary tree, or extrahepatic disease was observed. A CT-guided liver biopsy revealed a stromal mantle of spindle cells along with variable nests of cells on a desmoplastic fibrous background. There were also focal calcifications that resembled psammoma bodies. This was consistent with a desmoplastic epithelial tumor with calcified nested stroma (CNSET). The patient was seen for surgical evaluation by Transplant and Hepatobiliary Surgery.

**Fig. 2:**
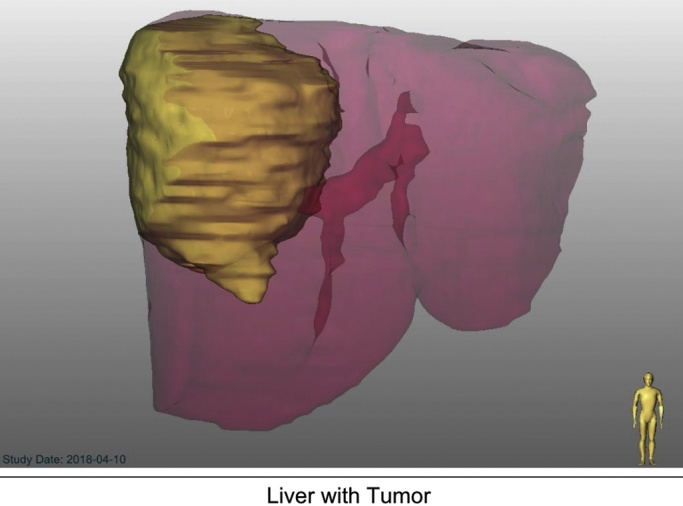
3D Rendering of patient’s liver with tumor utilized to estimate volume of remnant liver following potential resection.

Two options for surgical resection were discussed. The first, a single stage right trisectionectomy due to possible involvement of the middle hepatic vein, would be the preferred approach as it is a single operation and the potential for regeneration is high given the patient’s age. The second option would require two stages and would be utilized if the first option were not feasible based on the size of the FLR. The second option would require portal vein embolization (PVE) or ALPPS.A volumetric rendering of the patient’s liver and mass (MeVis scan) were obtained in order to best approximate residual hepatic parenchyma following resection of the mass (Fig. 2). Based on the scan, it was estimated that greater than 40 % of functional liver remnant would remain following right trisectionectomy. Ultimately, the decision of whether to pursue a single or two-stage procedure would be decided during the operation based on the tumor’s relation to the middle hepatic vein and how much residual functional parenchyma would remain post-resection.

**Fig. 3:**
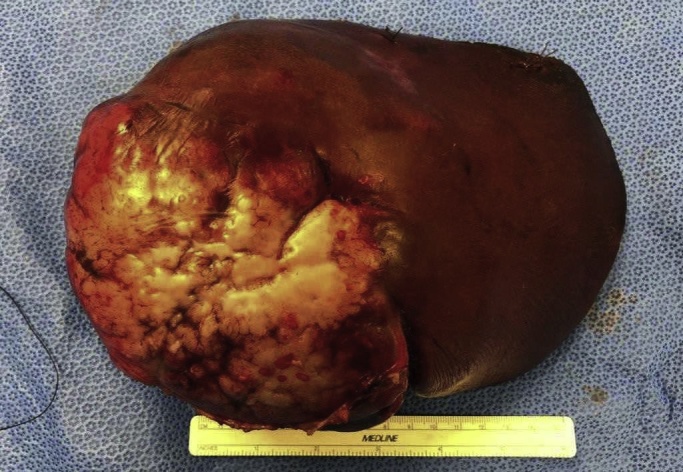
Intraoperative photograph of the excised CNSET.

Upon exposure via Muakuchi incision, the liver was found to be of normal color and normal surface with sharp edges and no signs of fibrosis. The rest of the abdomen had no evidence of tumor burden. Following mobilization of the right and caudate lobes of the liver from the inferior vena cava, intraoperative ultrasound allowed for identification of the tumor in contact with the middle hepatic vein occupying a large portion of the right side of the liver. It was estimated that the patient would have greater than approximately 40 % of her functional liver parenchyma remaining given the volume occupied by the non-functional tumor. Thus, a right trisectionectomy of segments 1, 4, 5, 6, 7, 8 was completed to surgically resect the tumor and a two stage approach was not necessary. The tumor measured 15.0 × 12.6 × 10 cm with negative margins and no extracapsular extension (Fig. 3). She received 3 liters of crystalloid and had approximately 200 mL blood loss. Her post-op course was uncomplicated and she was discharged on post-operative day (POD) six. The pathology report was consistent with CNSET. The patient returned to her normal active life and has had no evidence of recurrence more than a year post resection.

## 3 Discussion

CNSET are extremely rare, large, indolent tumors of the liver of uncertain cellular origin. They have distinct histopathological appearance and a relatively invariable appearance on imaging studies. It is a nonhepatocytic and nobiliary tumor of the liver of uncertain histogenesis with no normal cellular counterpart. CNSET has been called by several other names in the literature, such as ossifying malignant mixed epithelial and stromal tumor, ossifying stromal-epithelial tumor, and desmoplastic nested spindle cell tumor [7]. Patients are often asymptomatic with an incidentally found tumor. There have been several cases of patients presenting with abdominal pain, mass, or Cushing syndrome. Liver function tests are most often normal without elevation of aminotransferases, bilirubin, or alkaline phosphatase [1]. Levels of tumor markers are similarly normal.

These tumors display consistent radiographic findings. They are large, well circumscribed, heterogenous masses that have dense calcification. This calcification varies from being located centrally to diffusely throughout the tumor [8]. The differential diagnosis for CNSET includes hepatoblastoma, desmoplastic small round cell tumor, synovial sarcoma, and other metastatic possibilities [7,8]. The diagnosis is confirmed via biopsy.

CNSET affects females to males in a ratio of 2.5:1 and most frequently involves the right hepatic lobe (65 % of cases) [1]. This tumor, as evidenced by review of the cases reported, appears to have a low malignant potential overall [9]. Treatment involves surgical resection of the tumor via partial hepatectomy in a single or two-stage procedure depending on the size and location of the mass or liver transplantation if surgical resection is not possible. Two-stage approach via portal vein embolization (PVE) or ALPPS is necessary to optimize the FLR. ALPPS is preferred to PVE as it accelerates hypertrophy and has a greater probability of providing adequate hypertrophy. Patients who presented with Cushing syndrome have resolution of their symptoms with excision [3]. Recurrence has occurred in cases where there has been incomplete excision of the tumor [7]. Thus, complete excision of the tumor is critical. If there will be insufficient residual liver remnant following hepatectomy, another option such as ALPPS must be considered.

While a right trisectionectomy in a single operation was utilized in this case, an ALPPS was strongly considered due to the size of the tumor and concern for the amount of residual liver parenchyma following resection. This is a concern for an inadequate future liver remnant resulting in posthepatectomy liver failure after any liver resection. In cases where hepatectomy would result in an insufficient amount of remaining liver parenchyma, the options for staged hepatectomy include ALPPS, portal vein embolization and hepatectomy, and two-stage hepatectomy. ALPPS combines portal vein ligation and transection of the liver between the now deportalized portion and the planned remnant [10]. The benefit of this procedure is that it allows for hypertrophy of the residual remnant 80 % faster than other procedures [11]. Portal vein embolization and hepatectomy is reported to result in failure of curative liver resection in approximately 20 % of patients due to disease progression or insufficient hypertrophy.

As there are few reported cased of CSNET, the optimal therapeutic approach is not standardized. Though the origin of these tumors is not known, the risk of recurrence is low with negative margins based on published reports. It is clear that surgical resection of these masses is the current treatment of choice if there is an adequate future liver remnant.

## Sources of Funding

No direct funding sources to report.

## Ethical approval

As a case report, this article does not meet DHHS definition of research and thus does not require review by our institution’s IRB. Efforts were made by all authors to ensure compliance with HIPAA requirements.

## Consent

Written informed consent was obtained from the patient's parents for publication of this case report and accompanying images. A copy of the written consent is available for review by the Editor-in-Chief of this journal on request.

## Author contribution

Nicholas Olin: primary author

Ankit Patel: writer and editor

Susan S. Baker: editor, study design

Roberto Hernandez-Alejandro: study design/concept initiator

## Registration of research studies

N/A.

## Guarantor

Nicholas Olin

Ankit Patel

Roberto Hernandez-Alejandro

## Provenance and peer review

Not commissioned, externally peer-reviewed

## Declaration of Competing Interest

No conflicts of interest.
